# HER2 as a treatment target in desmoplastic small round cell tumors (DSRCT): a retrospective study in a certified sarcoma center

**DOI:** 10.1007/s00432-026-06668-6

**Published:** 2026-11-13

**Authors:** Christoph K. W. Deinzer, Katrin Benzler, Lars Zender, Ruth Ladurner, Robert Rottscholl, Jens Strohäker

**Affiliations:** 1 Department of Internal Medicine VIII - Medical Oncology and Pneumology, University Hospital Tübingen, Tübingen, Germany; 2 Center for Soft Tissue Sarcomas, GIST and Bone Tumors (ZWS) of the University Hospital Tübingen and the Comprehensive Cancer Center (CCC) Tübingen-Stuttgart, Tübingen, Germany; 3 Department of General, Visceral and Transplantation Surgery, University Hospital Tübingen, Tübingen, Germany; 4 Department of Pathology and Neuropathology, University Hospital Tübingen, Tübingen, Germany

**Keywords:** Desmoplastic small round cell tumors, DSRCT, Specialized sarcoma center, HER2, HER2 antibody–drug-conjugates (ADCs), Trastuzumab deruxtecan

## Abstract

**Purpose:**

Desmoplastic small round cell tumors (DSRCT) are exceptionally rare and aggressive sarcomas with limited therapeutic options and scarce clinical evidence. We aimed to characterize the clinical course and treatment of patients with molecularly confirmed DSRCT and to investigate HER2 expression as a potential therapeutic target, including real-world experience with trastuzumab deruxtecan (T-DXd).

**Methods:**

We retrospectively analyzed patients with DSRCT treated at our specialized and certified sarcoma center. Diagnosis was confirmed by detection of the pathognomonic EWSR1::WT1 fusion. Clinical characteristics, treatment strategies, surgical outcomes, HER2 immunohistochemistry (IHC), and fluorescence in situ hybridization (FISH) results were assessed. Clinical outcomes of patients receiving T-DXd were reviewed.

**Results:**

Eleven patients were identified (median age 22 years, range 5–63; 81.8% male). HER2 IHC was 0 in three patients (27.3%), 1 + in three (27.3%), and 2 + in five (45.5%); none showed 3 + expression, and all tumors were FISH-negative. Two extensively pretreated patients with HER2 IHC 2+/FISH-negative tumors received off-label treatment with T-DXd and demonstrated initial radiological responses. One patient experienced subsequent progression and died, whereas the second maintained disease control after more than 10 cycles despite chronic dialysis and without substantial additional toxicity.

**Conclusions:**

This molecularly confirmed cohort highlights the aggressive clinical course and substantial unmet therapeutic need in DSRCT. HER2 protein expression was observed in a considerable proportion of tumors despite the absence of HER2 amplification. Responses to T-DXd in two HER2 IHC 2+/FISH-negative patients provide a hypothesis-generating clinical signal supporting further investigation of HER2-directed antibody–drug conjugates in DSRCT.

## Introduction

Desmoplastic small round cell tumors (DSRCT) are aggressive and extremely rare mesenchymal malignancies with an incidence of 0.2–0.5 cases/million per year (Lettieri et al 2014), which was firstly described in literature by Gerald and Rosai in 1989 (Gerald & Rosai 1989) and which is one entity of more than 100 subtypes of soft tissue tumors in the current WHO classification (Editorial Board 2020). Accordingly, the management of patients with DSRCT, in line with current recommendations for soft tissue sarcomas, should be performed at specialized sarcoma reference centers (Calukovic et al 2025; Gronchi et al 2021).

From a histopathological perspective, DSRCT is characterized by nests of small round blue cells embedded within a prominent desmoplastic stroma (Gerald et al 1991). The tumor is molecularly characterized by the unique and pathognomonic chromosomal translocation t(11;22) (p13;q12) leading to a fusion of Ewing sarcoma gene (EWS) and Wilms’ tumor gene (WT1). The EWSR1::WT1 fusion as the driver of DSRCT changes gene expression and consequently leads to tumor growth (Dufresne et al 2012; Ladanyi and Gerald 1994). DSRCT occurs four times more often in males than in females (male/female ratio 4:1) and the patients are in the mean 22 years old at time of diagnosis with a range of 6 to 49 years (Dufresne et al 2012; Gerald et al 1998). Prognosis of DSRCT is poor with 5-year overall survival of only around 15–30% (Hayes-Jordan et al 2016).

Clinically, 90% of DSRCT primarily develop in the abdominal cavity and pelvis and involve liver, lung, lymph nodes and bones secondarily. DSRCT is often diagnosed in advanced stages with ascites, abdominal pain, bowel obstruction, vomiting and weight loss because of absent or minor symptoms in the beginning stages of the disease (Bulbul et al 2017; Hendricks et al 2021).

Primary therapeutic approach following diagnosis is systemic chemotherapy integrated into a multimodal treatment strategy that may include cytoreductive surgery and/or radiotherapy. Owing to the limited number of patients and the extended time spans over which data have been collected, up to 30 distinct chemotherapy regimens have been reported in the literature for the treatment of DSRCT. The regimens most frequently administered in a neoadjuvant or palliative setting are largely extrapolated from established protocols used in Ewing sarcoma or soft tissue sarcoma, including, among others, VIDE, P6, VDC/IE, VAI, VAC, and CEVAIE (Hendricks et al 2021). In the second-line setting, therapeutic options include high-dose ifosfamide as well as combination regimens such as temozolomide plus irinotecan or cyclophosphamide plus topotecan (Jeong et al 2021). Patients demonstrating chemosensitivity were more likely to derive benefit from subsequent cytoreductive surgery, whereas disease progression during induction chemotherapy is generally considered a contraindication to surgical intervention (Hendricks et al 2021; Subbiah et al 2018). In patients without extraperitoneal metastases (EPM), a multimodal therapeutic approach comprising systemic chemotherapy, complete macroscopic resection, and postoperative whole-abdominopelvic radiotherapy (WAP-RT) may facilitate prolonged survival. In contrast, no survival benefit from surgical intervention has been demonstrated in patients presenting with EPM. Furthermore, the therapeutic value of adjunctive hyperthermic intraperitoneal chemotherapy (HIPEC) remains to be conclusively established (Hayes-Jordan et al 2015; Hendricks et al 2021; Honore et al 2015). In the pursuit of targeted therapeutic strategies for patients with extraperitoneal metastases (EPM), several tyrosine kinase inhibitors (TKIs), including imatinib, sorafenib, and sunitinib, have been evaluated in clinical studies. However, imatinib had no significant clinical benefit (De Sanctis et al 2017; Hendricks et al 2021) and sunitinib showed only limited efficacy (Italiano et al 2013). Notably, apatinib and anlotinib have shown symptomatic improvement and progression-free survival exceeding four months in isolated case reports (Chen and Feng 2019; Shi et al 2018).

In search of new immunotherapeutic targets, Zhang et al. described an immunohistochemical HER2 protein expression in 39% (7/18; 11: negative; 4: 1 + ; 2: 2 + ; 1: 3 + ) of the examined DSRCT samples, although predominantly at low levels in most of the positively stained tumors (Espinosa-Cotton and Cheung 2021; Zhang et al 2003). Espinosa-Cotton et al. could verify a surface expression of HER2 on DSRCT cell lines. Notably, a HER2 x CD3 T cell-engaging bispecific antibody was able to achieve complete regression of established tumors in an intraperitoneal DSRCT mouse model (Espinosa-Cotton et al 2023). In another study, treatment with the antibody–drug conjugate trastuzumab deruxtecan (T-DXd) induced tumor regression in HER2-expressing DSRCT patient-derived xenografts, cell lines, and organoid models (Zhang et al 2024). In the clincial study “DESTINY-PanTumor 02”, T-DXd, which is directed against the human epidermal growth factor 2 (HER2) and a potent topoisomerase I inhibitor, was evaluated in various solid tumors, including endometrial, cervical, ovarian, pancreatic, biliary tract, and bladder cancers. The study demonstrated sustained clinical benefit, notable survival outcomes, and a safety profile consistent with prior reports. The most pronounced therapeutic effect was observed in the immunohistochemistry (IHC) 3 + population (Meric-Bernstam et al 2024). DSRCTs and other soft tissue sarcomas were not included in this study; therefore, the efficacy of T-DXd in DSRCT has not yet been assessed in a multicenter, prospective clinical trial and, due to the rarity of the disease, is unlikely to be evaluated in such a setting in the future.

Beyond the limited in vitro data, evidence for the efficacy of T-DXd in DSRCT is currently confined to a few isolated case reports and a small case series involving three patients (Brahmi et al 2025; Chalasani et al 2009; Donze et al 2025). Moreover, HER2 expression in DSRCT remains poorly characterized, and consequently, assessment of HER2 status and the use of antibody–drug conjugates (ADCs) in DSRCT have not yet been established, even within certified sarcoma centers.

Accordingly, the objective of the present study is to provide an analysis of HER2 expression in a real-word cohort of DSRCT patients and to report about the T-DXd efficacy in DSRCT patients treated at a certified (by the German Cancer Society) sarcoma center, with the ultimate goal of expanding the therapeutic arsenal against this rare and complex tumor entity.

## Methods

### Inclusion and exclusion criteria

All patients who met the following inclusion criteria were eligible for our study:

- Treatment of DSRCT at the certified Sarcoma Center of the University Hospital Tübingen and the Comprehensive Cancer Center (CCC) Tübingen-Stuttgart between 2013 and 2025.
- Availability of formalin-fixed, paraffin-embedded (FFPE) DSRCT samples in the Institute of Pathology of the University Hospital Tübingen.

**Fig. 1:**
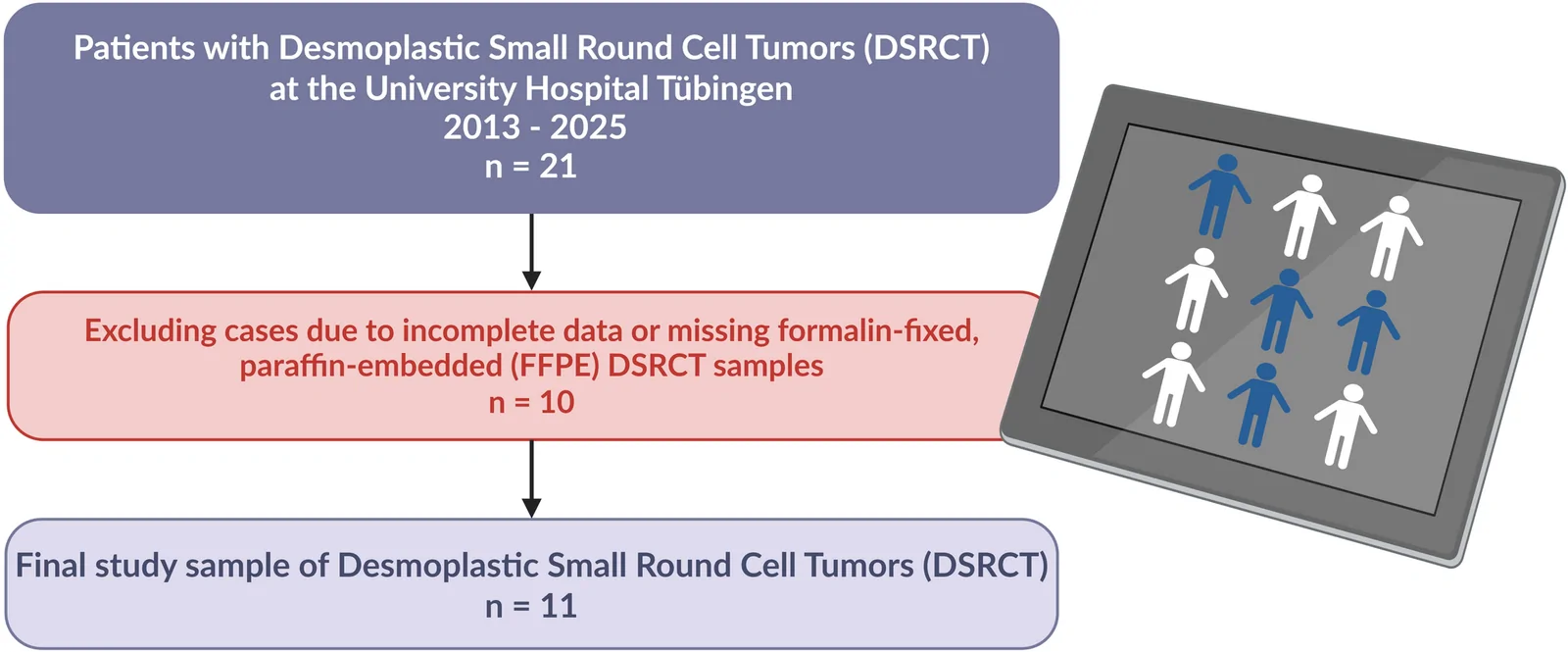
Schematic overview of the study population selection

Patients with incomplete data, missing FFPE samples or without confirmed histology of DSRCT were excluded as depicted in Fig. 1.

Figure is created in BioRender (Deinzer 2026).

### HER2 immunohistochemistry and FISH analysis

The HER2-status was assessed according to local standards by using a validated assay using HER2 antibody (Clone 4b5, Roche Diagnostics International, Rotkreuz, Switzerland) on FFPE slides on a Benchmark XT (Roche Diagnostics). Expression of HER2 was scored on a scale from 0 to 3+. Tumors with a score of 3 + were considered HER2-positive. HER2 amplification was determined by fluorescence in-situ hybridization (FISH) using the Zytolight CEN17/ERBB2 dual color probe (Zytovision, Bremerhaven, Germany) for all cases. Both immunohistochemistry and FISH protocols can be provided for reproduction upon request.

### Clinical parameters

Patient data were obtained from electronic hospital information systems: Age at diagnosis, gender, histology, tumor localization, location of metastases, date of diagnosis, date of histology, date of relapse/progression, location of relapse, date of death, date of surgery, status of resection, chemotherapy regimens. Progression-free survival (PFS) was calculated as the time to relapse or time to progression after the first systemic therapy. Overall survival (OS) was calculated as the time from diagnosis to death.

### Statistical analysis and visualization

GraphPad Prism 9.4.1 (Dotmatics, Boston, MA, USA) and SPSS Statistics 31 (IBM, Armonk, NY, USA) were used for statistical analysis. Graphical illustrations were created using BioRender (BioRender.com, Toronto, ON, Canada).

## Results

Between 2013 and 2025, eleven patients with DSRCT could be included in the study. The pathognomonic EWSR1-WT1 fusion was proven in each case at the time of primary diagnosis. The median age at diagnosis was 22 years (range 5–63, interquartile range (IQR) 15–43). Of these the majority was male (n = 9, 81.8%). All patients presented with abdominal masses. At the initial diagnosis liver metatases were detectable in four patients (36.4%), bone metatases were present in two patients (18.2%) and intrathoracic manifestations in one patient (9.1%). Most patients underwent preoperative biopsy and neoadjuvant therapy with varying chemotherapy regimens. Preoperative chemotherapy was administered intravenously to ten of eleven patients (90.9%), with five patients (45.5%) receiving chemotherapy according to the Cooperative Soft Tissue Sarcoma Study Group (CWS) Protocol (ifosfamide, vincristin, actinomycin D, adriamycin, etoposide), three patients (27.3%) were treated with VDC/IE (vincristin, doxorubicin, cyclophosphamide, ifosfamide and etoposide) and VIDE (vincristin, ifosfamide, doxorubicin, etoposide) was administered to two patients (18.2%).

All patients underwent surgical exploration. Six patients (54.5%) had complete surgical resection with a completeness of cytoreduction (CC) Score 0 (CC-0) status and five (45.5%) had intraoperative HIPEC therapy with cisplatin and doxorubicin. Two patients (18.2%) underwent debulking surgery and three (27.3%) were aborted after exploration and biopsies were taken only. Depending on protocol and resection status postoperative chemotherapy was administered accordingly.

Five patients (45.5%) were never cancer free, four (36.4%) out of the six remaining patients recurred after a median of 19 months (range 1–47). Four patients (36.4%) are confirmed dead, four (36.4%) are confirmed to be in remission/stable disease. The remaining three patients (27.3%) have been lost to follow up with missing data mainly for being international patients with incomplete resections or progressive disease and are presumably dead. Due to missing follow-up data, these three patients were excluded from survival analysis.

**Fig. 2:**
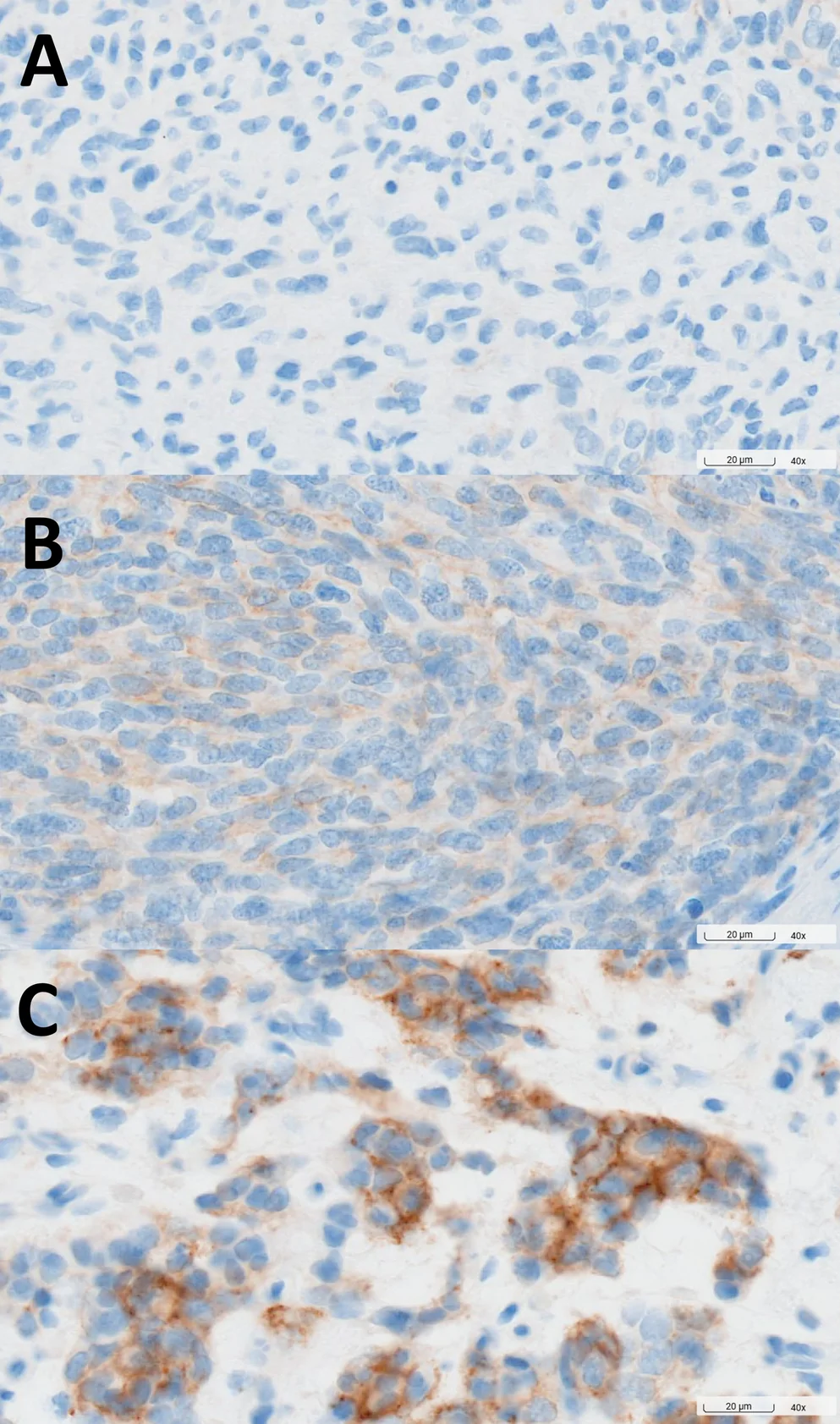
Representative photomicrographs of HER2 staining in resection specimen. **A**: no staining (HER2 score 0), 15-year-old male patient; **B**: weak staining (HER2 score 1 +), 17-year-old female patient; **C**: moderate staining (HER2 score 2 +), 10-year-old male patient

HER2 testing was performed on FFPE tissue of every patient. Three patients (27.3%) had negative HER2 immunohistochemistry (IHC), three were 1 + (27.3%) and the remaing five patients (45.5%) were 2+. No patient was 3+. All samples underwent FISH testing and all were negative for ampflication. Patient characteristics are summarized in Table 1 and representative photomicrographs of HER2 immunohistochemistry are depicted in Fig. 2.

**Table 1:** Characteristics of the DSRCT patient cohort

Characteristics	Total patient number 11 (100% of cohort)
Gender, n (%)	
Male	9 (81.8%)
Female	2 (18.2%)
Age (years), median (range; IQR)	22 (5–63; 15–43)
Staging according to TNM	
Tumor	
T1	0 (0%)
T2	3 (27.3%)
T3	1 (9.1%)
T4	7 (63.6%)
Lymphnodes	
N0	2 (18.2%)
N1	3 (27.3%)
Nx	6 (54.5%)
Metastases	
M0	4 (36.4%)
M1	7 (63.6%)
HER2 Immunohistochemistry (IHC)	
0	3 (27.3%)
1 +	3 (27.3%)
2 +	5 (45.5%)
3 +	0
All samples underwent FISH testing and all were negative for HER2 amplification.	
Perioperative chemotherapy	
CWS protocol	5 (45.5%)
VDC/IE	3 (27.3%)
VIDE	2 (18.2%)
Surgery	
Complete resection	6 (54.5%)
Debulking surgery	2 (18.2%)
Abortion of resection (after exploration and biopsies only)	3 (27.3%)
Progression-free survival (PFS)	
Number of patients	8
Events (progression)	6
Censored	2
Median PFS	15.0 months (95% confidence interval: 5,3; 24,7)
Overall survival (OS)	
Number of patients	8
Events (death)	4
Censored	4
Median OS	58.0 months (95% confidence interval: 38.9; 77.1)

**Fig. 3:**
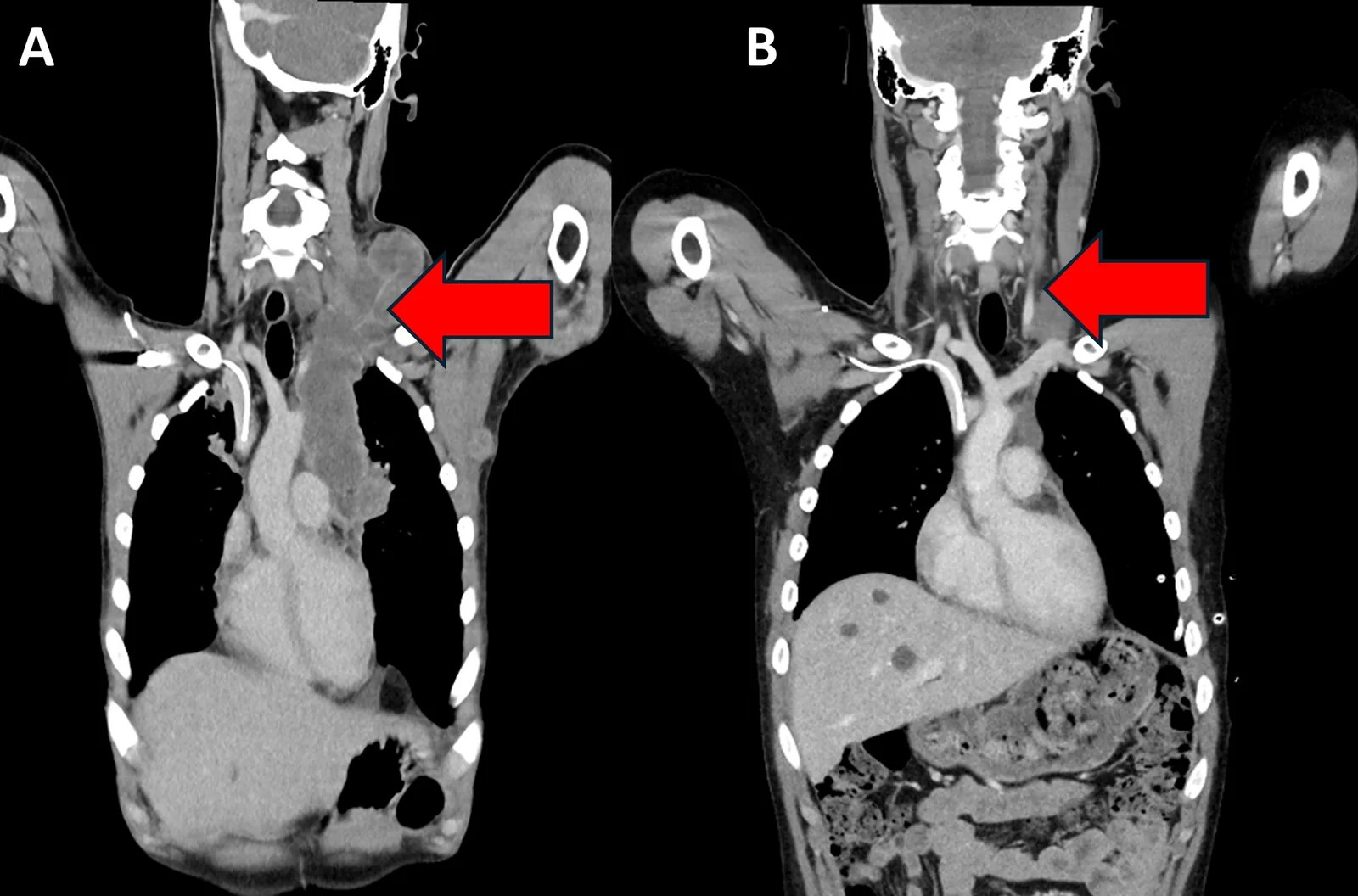
Representative computed tomography scans of thorax and upper abdominal region of a 18-year-old male. **A**: Baseline CT scan with lymph node metastases (red arrow), **B**: CT scan after three cycles of T-DXd with only small residual remnants of lymph node metastases (red arrow)

Trastuzumab deruxtecan treatment was given to two patients in our cohort. The first one was a 18-year-old male who had initially presented to an outside hospital in August of 2023 with very widespread disease including liver and bone metastasis as well as extensive peritoneal disease. He had responded well to 9 cycles of CWS treatment, was later switched to VDC/IE of which he had 8 cycles and then underwent Cytoreductive Surgery (CRS) combined with Hyperthermic Intraperitoneal Chemotherapy (HIPEC) with a resection at our sarcoma center achieving a completeness of cytoreduction (CC) Score 0 (CC-0; no visible tumor remaining in the abdominal cavity). Unfortunately, he had very early hepatic progress and was restarted on chemotherapy with VDC/IE for another two cycles. He progressed and was switched to Irinotecan/Temozolomid. At the following progress, he was switched to T-DXd 5.4 mg/kg body weight every three weeks intravenously in November 2025 after confirming his DSRCT was IHC 2 + for HER2. He had tremendeous response on his first imaging computed tomography scan in January 2026 after three cyles of T-DXd (see Fig. 3). However, he had mixed response with an overall increase of tumor burden in April 2026 particularly with pleural disease. Repeat sampling was done during a palliative pleurodesis and the tissue there was IHC 1 + for HER2 and again FISH-negative. The patient succumbed to his disease shortly after.

The other patient was diagnosed in September of 2023 as a then 43-year-old male with a large retroperitoneal and mesenteric mass as well as peritoneal disease. After a single cycle of Doxorubicin and Ifosfamide he was switched to 6 cycles of VDC/IE preoperatively, followed by four cycles postoperatively after laparotomy and incomplete resection. In June 2025, he went into chronic kidney disfunction with an eGFR (estimated Glomerular Filtration Rate) of around 8 ml/min/1.73m^2^, attributed to obstruction and treatment-related toxicity. He therefore continued with doxorubicin monotherapy for five cycles and was subsequently switched to Ipilimumab and Nivolumab for another three cycles, after which he experienced disease progression again. He started dialysis in December of 2025 and progressed again in January of 2026 and was switched to T-DXd 5.4 mg/kg body weight every three weeks shortly after. He responded well on his first scan and continues to respond as of August 2026. Despite being on chronic renal replacement therapy, he has not experienced any significant toxicities after more than 10 cycles of T-DXd and no dose modifications or treatment interruptions have been necessary to date.

**Fig. 4:**
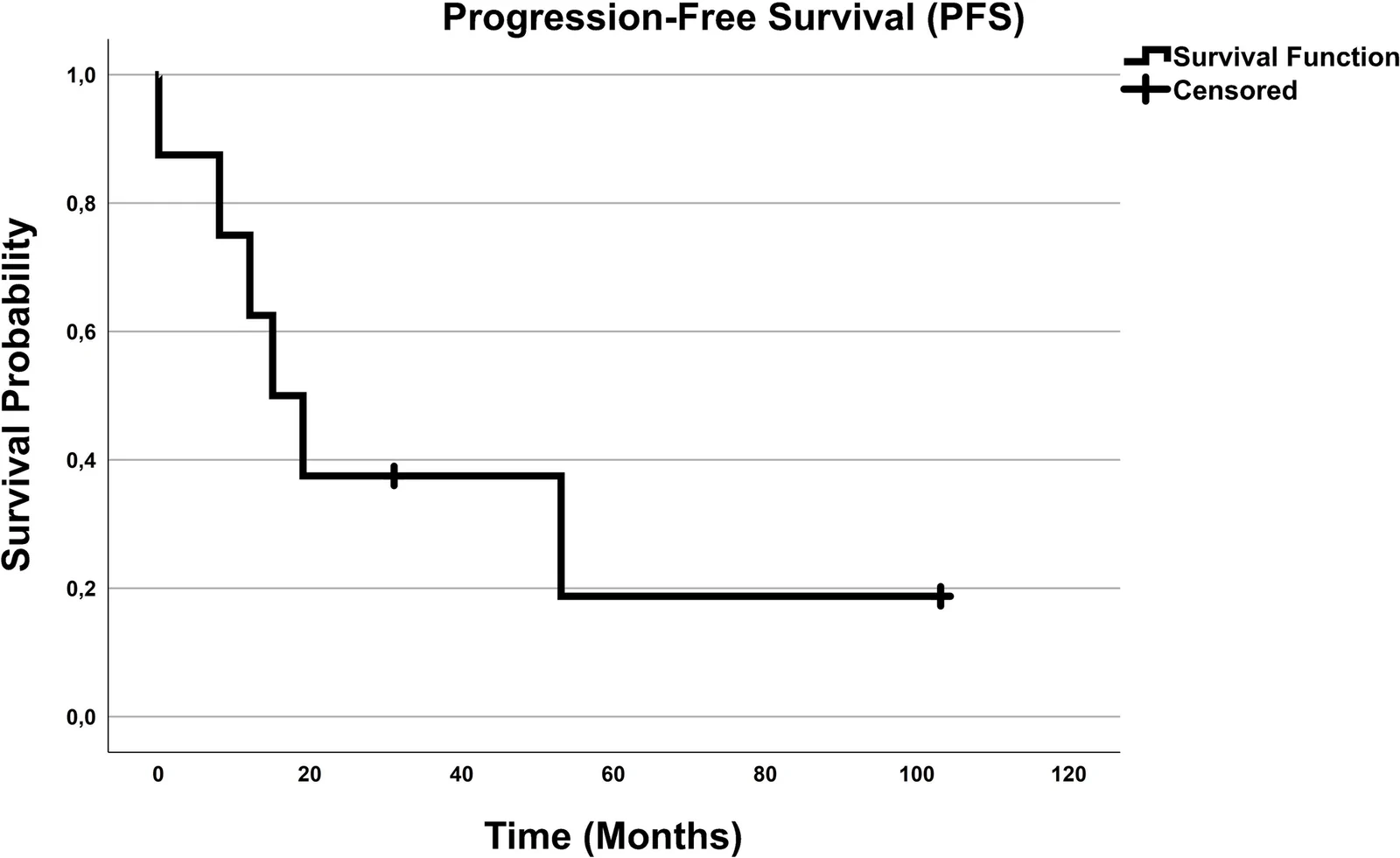
Kaplan–Meier estimates of progression-free survival

**Fig. 5:**
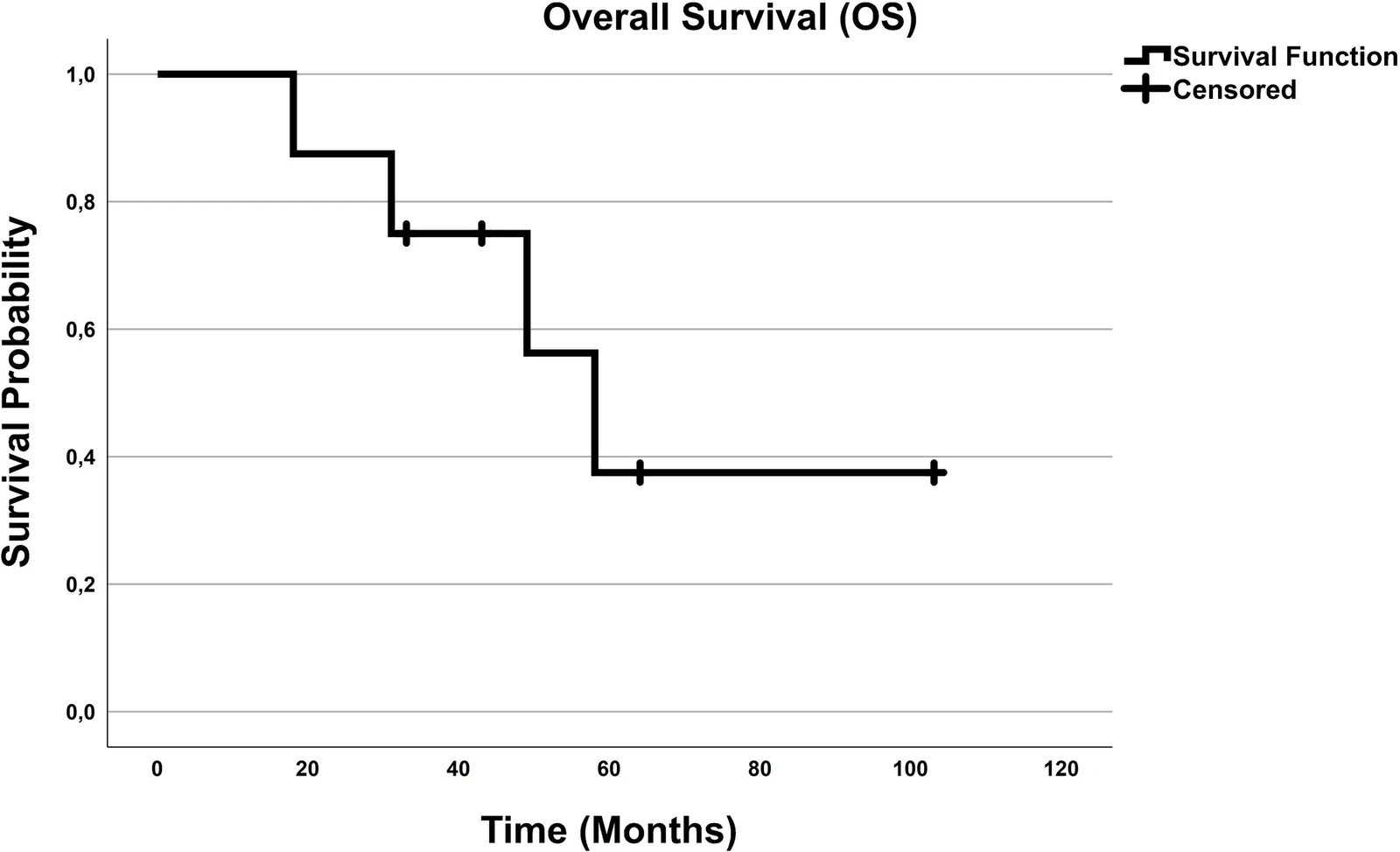
Kaplan–Meier estimates of overall survival

Kaplan–Meier analysis of the eight evaluable patients demonstrated a median progression-free survival (PFS) of 15.0 months and a median overall survival (OS) of 58.0 months in our cohort. These results are depicted in Figs. 4 and 5.

## Discussion

DSRCTs are exceedingly rare sarcomas with an incidence of 0.2–0.5 cases/million per year (Lettieri et al 2014). The disease predominantly affects teenagers and young adults with a male to female ratio of 4:1 (Dufresne et al 2012; Gerald et al 1998). Due to its rarity, evidence guiding systemic and local treatment remains limited and is predominantly derived from retrospective series, institutional experiences, and treatment strategies adapted from other sarcoma entities. In this context, our cohort provides clinically relevant real-world data from a specialized and certified sarcoma center, comprising 11 patients with molecularly confirmed DSRCT. Despite the limited absolute number of patients, which reflects the rarity of the disease rather than limited observation, the cohort provides a detailed characterization of disease presentation, multimodal treatment, surgical outcomes, HER2 expression, and subsequent treatment trajectories. In addition, our experience with trastuzumab deruxtecan (T-DXd) in two heavily pretreated patients provides a clinically relevant signal warranting further investigation.

The demographic and clinical characteristics of our cohort are consistent with the previously described phenotype of DSRCT, with a marked male predominance and a young age at diagnosis. The median age was 22 years, and 81.8% of patients were male. All patients presented with an abdominal mass, while metastatic disease was already present in 63.6% at diagnosis. Liver metastases were observed in 36.4%, bone metastases in 18.2%, and intrathoracic manifestations in 9.1% of patients. Furthermore, 63.6% of tumors were classified as tumor stage T4. These findings illustrate the advanced stage at which DSRCT commonly presents and the substantial tumor burden that frequently characterizes this disease. They also emphasize the importance of early diagnosis and referral to specialized sarcoma centers with multidisciplinary expertise.

Given this disease is frequently diagnosed at advanced stages most sarcoma specialists will favor a multimodal treatment of preoperative chemotherapy, followed by complete cytoreductive surgery, intraoperative HIPEC, postoperative adjuvant chemotherapy and radiation (Hendricks et al 2021). There are currently no guidelines from any of the established medical or surgical oncolgy societies for the treatment of DSRCT. The median overall survival for DSRCT patients averages 26 months with a 5-year overall survival rate of < 20% (Gani et al 2019). VDC/IE is a potent yet intense chemotherapy regimen that was reported to have a higher than 50% objective response rate in DSRCT (Subbiah et al 2018). The same study by Subbiah et al. described 186 patients from MD Anderson Cancer Center, Houston, Texas, whose second line chemotherapy was quite variable with temozolimide/irinotecan, high dose ifosfamide and cyclophosphamide/topotecan being their most common choices, albeit with limited effects (Subbiah et al 2018). Well tolerated and effective treatment options are sought after for this rare tumor type. Treatment in our cohort reflected the multimodal approach generally pursued for DSRCT but also demonstrated considerable heterogeneity. Ten of eleven patients received intravenous neoadjuvant chemotherapy, using regimens based on the CWS, VDC/IE, or VIDE protocols. The use of intensive chemotherapy regimens reflects the absence of a universally accepted systemic treatment standard for DSRCT and the historical influence of Ewing sarcoma treatment strategies. However, the heterogeneity observed in our cohort also illustrates the difficulty of establishing a uniform treatment algorithm in an exceptionally rare disease. Treatment decisions are frequently individualized according to age, disease extent, response to systemic therapy, and the feasibility of subsequent cytoreductive surgery.

Surgical treatment represented another major challenge. Although all patients underwent surgical exploration, complete macroscopic resection (CC-0) was achieved in only six patients (54.5%). Two patients underwent debulking surgery, whereas resection was abandoned after exploration and biopsy in three patients because complete cytoreduction was not feasible. These findings reflect the frequently extensive intra-abdominal and peritoneal involvement of DSRCT and underline the importance of multidisciplinary treatment planning. In selected patients, cytoreductive surgery combined with HIPEC may provide an opportunity for substantial reduction of tumor burden. However, the role and optimal selection of patients for such approaches remain difficult to define in the absence of prospective comparative data.

A central finding of the present study was the systematic evaluation of HER2 expression in all tumors. HER2 IHC was negative (0) in three patients, 1 + in three, and 2 + in five patients, while no tumor showed 3 + expression. Importantly, all tumors were negative for HER2 amplification by FISH including the cases with Score 2+. Thus, 45.5% of the tumors demonstrated intermediate HER2 protein expression (HER2 score 2 +) in the absence of detectable HER2 gene amplification. This finding is clinically relevant in view of the increasing recognition that antibody–drug conjugates may exert antitumor activity in tumors with HER2 expression that would traditionally not have been classified as HER2-positive based on amplification or strong protein overexpression. Historically, trastuzumab as an Anti-ERB2 antibody played little to no role in the treatment of DSRCT since it only showed anti-cancer activity in patients with 3 + HER2 expression or HER2 amplification. Data on HER2 expression in sarcomas is scarce. In the early 2000 s Scotlandi et al. reported on HER2 in osteosarcoma and Ewing sarcomas and described HER2 protein expression (3 + and 2 + ) in 32% and 16% respectively (Scotlandi et al 2005). For sarcomas in general, HER2 protein expression (3 + and 2 +) was reported to be around 10% (Foster et al 2003). Zhang et al. reported a immunohistochemical HER2 protein expression (HER2 score 1+, 2 + and 3 +) in 39% of DSRCT samples, which was lower than in our patient cohort, where 73% of our patients had a HER2 score of 1 + or higher (Zhang et al 2003).

With the FDA approval of T-DXd for HER2 low breast cancers following the results of the “DESTINY-Breast04” (Modi et al 2025) trial and the promising results of “DESTINY-PanTumor 02” (Meric-Bernstam et al 2024) T-DXd has also become an interesting agent in the treatment for selected sarcoma patients. The recent European Commission tumor agnostic approval of trastuzumab deruxtecan (T-DXd) monotherapy in June 2026 for the treatment of previously treated, unresectable or metastatic HER2-positive (IHC 3 + ) solid tumours with no satisfactory alternative treatment options highlights the increasing clinical relevance of HER2 as a therapeutic target across tumour entities. However, the current indication in Europe is restricted to tumours with HER2 IHC 3 + expression (Daiichi-Sankyo 2026; EMA 2026).

Brahmi et al. recently published data on the use of T-DXd in three patients with DSRCT. They measured ERBB2-RNA in samples from 13 adult DSRCT patients. All of them showed marked elevation of RNA, indicating that T-DXd may be a potential treatment option for all of them. All three patients they had treated with T-DXd showed clinical response as well as partial response on imaging (Brahmi et al 2025). A research group from Memorial Sloan Kettering Cancer Center, New York, USA, published data on 19 patients receiving T-DXd. They showed that IHC results—depending on the antibodies used—failed to predict RNA levels or treatment response. They reported also one early progression after 15 weeks as well as two patients progressing after around 30 weeks and one after a year. Eleven patients in their cohort were responding ongoingly at the time of publication after more than 6 months of treatment (Slotkin et al 2026).

Of particular interest, two patients in our cohort also received T-DXd after extensive pretreatment. Both tumors showed HER2 IHC 2 + expression without HER2 amplification. Both patients demonstrated an initial clinical and radiological response following treatment with T-DXd, despite having progressive disease after multiple previous systemic treatment strategies. These observations are notable because they suggest that HER2-directed therapy may have activity in DSRCT even in the absence of HER2 amplification. However, the two cases should be regarded as hypothesis-generating observations rather than evidence of treatment efficacy. The small number of treated patients, absence of a comparator, and potential for selection bias preclude any estimate of response rate or treatment effect.

The observation of responses in two HER2 IHC 2+/FISH-negative tumors is particularly relevant in light of the mechanism of action of T-DXd. Unlike conventional HER2-directed therapies, antibody–drug conjugates may retain activity in tumors with lower levels of HER2 expression, potentially owing to efficient intracellular delivery of the cytotoxic payload and a bystander effect. Consequently, HER2 gene amplification may not be an obligatory prerequisite for therapeutic activity. Nevertheless, the present data do not demonstrate that HER2 IHC 2 + status is a predictive biomarker for T-DXd in DSRCT. Rather, they provide a rationale for prospective evaluation of this therapeutic strategy and for more systematic investigation of the relationship between HER2 expression and treatment response.

The difference in HER2 expression between the initial and subsequent pleural tumor sample in the first patient further emphasizes the potential relevance of tumor heterogeneity. Whether the reduction from IHC 2 + to 1 + represents true biological evolution, selection of a HER2-low subclone under treatment pressure, spatial heterogeneity, or analytical variation cannot be determined from the available material. Future studies should therefore consider assessing HER2 expression across different metastatic sites and, where clinically feasible, at different time points during treatment. Quantitative assessment of HER2 expression and integration with molecular profiling may further help to determine whether HER2 heterogeneity contributes to primary or acquired resistance to T-DXd.

An important strength of this cohort is the molecular homogeneity of the included cases. The EWSR1::WT1 fusion was confirmed in all 11 patients, providing a robust molecular definition of DSRCT. This is particularly relevant for a rare tumor in which historical series may have been affected by diagnostic heterogeneity. The consistent molecular confirmation strengthens the interpretation of the clinical and HER2 findings and supports their attribution to molecularly defined DSRCT.

Several limitations need to be acknowledged. First of all, the cohort size was small. However, this limitation needs to be considered in the context of the rarity of DSRCT. In a rare sarcoma, an 11-patient cohort from a single specialized sarcoma center represents a meaningful accumulation of clinical experience and may provide information that is difficult to obtain otherwise. Nevertheless, the sample size precludes meaningful statistical inference, and the reported percentages and Kaplan–Meier estimates should not be interpreted as population-level estimates but rather as hypothesis-generating observations that may inform the design of future clinical trials. Furthermore, the retrospective nature of the analysis, heterogeneous chemotherapy regimens, absence of a control group, and incomplete follow-up limit conclusions regarding comparative treatment efficacy and long-term outcomes. In particular, the T-DXd experience is limited to two patients and cannot establish efficacy or safety and should be considered hypothesis-generating. Finally, HER2 testing was performed by IHC with confirmatory FISH in all cases, and the number of available samples was too small to assess the prognostic or predictive significance of HER2 expression.

Despite these limitations, the present data are clinically relevant because they address an important unmet need in an exceptionally rare malignancy. The value of a rare-disease cohort should not be assessed solely by absolute patient numbers but also by the proportion of the available clinical experience that can be systematically characterized. Our cohort combines molecular confirmation of all cases with detailed treatment and surgical data, systematic HER2 assessment, and longitudinal observation of patients receiving T-DXd. Particularly, the observation of responses in two extensively pretreated patients with HER2 IHC 2+/FISH-negative tumors provides a clinically meaningful signal that merits further investigation.

Given the rarity of DSRCT, prospective randomized trials specifically dedicated to this entity will be challenging to conduct. International multicenter collaborations, prospective registries, basket trials, and molecularly or biomarker-defined studies may therefore represent more feasible approaches for evaluating novel therapies. Such studies should prospectively characterize HER2 expression, ideally across multiple disease sites, and correlate quantitative HER2 levels with response, duration of benefit, and mechanisms of resistance. The present observations provide a rationale for considering DSRCT in future clinical studies of HER2-directed antibody–drug conjugates.

In conclusion, our cohort of 11 molecularly confirmed DSRCT patients demonstrates the aggressive clinical course of this disease and the substantial challenges associated with achieving durable disease control despite intensive multimodal treatment. Systematic HER2 assessment identified protein expression in a considerable proportion of tumors, although none demonstrated HER2 amplification. Importantly, two extensively pretreated patients with HER2 IHC 2+/FISH-negative tumors experienced initial responses to T-DXd, with one patient achieving ongoing disease control beyond 10 treatment cycles despite chronic dialysis. To the best of our knowledge, only limited data have been published to date on the use of T-DXd in patients undergoing hemodialysis. These observations do not establish the efficacy of T-DXd in DSRCT but T-DXd may outperform other second-line treatments, for which median progression-free survival is typically less than 6 months (Mello et al 2021) and could provide a clinically relevant hypothesis-generating signal and support further prospective investigation of HER2-directed antibody–drug conjugates in this rare and therapeutically challenging sarcoma subtype.

## Data Availability

The datasets generated and analyzed during the current study are not publicly available due to patient data protection and privacy but are available from the corresponding author on reasonable request.

## Abbreviations

ADC: Antibody-drug conjugate​
CRS: Cytoreductive surgery
CWS: Cooperative soft tissue sarcoma study group
DSRCT: Desmoplastic small round cell tumor
EPM: Extraperitoneal metastases
FFPE: Formalin-fixed, paraffin-embedded (FFPE)
HER2: Human epidermal growth factor 2
HIPEC: Hyperthermic intraperitoneal chemotherapy
IHC: Immunohistochemistry
IQR: Interquartile range
T-DXd: Trastuzumab deruxtecan
